# Medicus Nascitur, Non Fit

**Published:** 1891-09-19

**Authors:** 


### "MEDICUS NASCITUR, NON FIT."

A few years ago there was an extensive correspondence carried on in some of the daily papers under the heading, "What to do with Our Boys"; and although, perhaps, much importance cannot be attached to such an expression of opinion, one could not fail to be struck with the almost complete absence of one most important point in this question, viz., "What our boys *wish* to be done with." This point is no doubt recognised to a much greater extent than it was some years ago, but still not sufficiently, for in a quite recent publication, a guide to the dental profession, the introduction is addressed to those who intend "to make their sons dentists."

With regard to the medical profession, it will at once be obvious that a deep interest in the work is most essential to the student if he is to attain the least degree of success; yet, strange to say, a large number of youths are annually "put" into the profession with most unfortunate results. We do not mean to say that these young men are entered in direct opposition to their own wishes, for nowadays such cases must be extremely rare, but that they have not shown any inclination or desire to become doctors. Those who are responsible for this state of things will tell you that their sons are not old enough to know their own minds, that the medical profession is as good as any other, that *they will get to like it*, and that once qualified they can always obtain a living anywhere.

We cannot deal here with the status of the profession or its profitableness, but it will be obvious that the only chance of real success will depend on their getting to like it, and on this chance practical men and men of business will speculate the large sum of money necessary for medical education, when they would not risk the tenth part in some of the finest investments in the money market. It will be our endeavour to show that those entering the profession under the circumstances indicated above are in the great majority of cases unsuccessful, and, in fact, form the bulk of failures from all causes. We have obtained statistics upon this point by personally eliciting from a large number of senior students and young qualified men certain facts regarding their entry into the profession, their academical career, and their present status. There has not been the slightest attempt to select our cases in any way, but they have been gathered more or less at random as we happened to be brought in contact with them, and are mostly gleaned from two or three of the large metropolitan schools. There are some unavoidable errors to be taken into consideration; thus a successful man would certainly be more likely to think that it was solely by his own wish that he entered the profession, whilst an unsuccessful one would probably think the reverse. There is, however, another error which tells in the opposite direction, namely that a large number of those who are "put" in do not stay more than a year or two, and in our statistics we do not include any student of less than four years' standing. We believe that this latter consideration more than counterbalances the former, but in any case the following figures must be fairly conclusive. The average academical year of those taken was from the sixth to seventh, and of the whole number about 45 per cent. were fully qualified. We notice in the first place that rather more than 30 per cent. were entered without any particular inclination on their part. We find that of the whole number 16 per cent. may be said to have done very well, 24 per cent. well, 20 per cent. fairly well, and 40 per cent. badly. Of the unfortunate 30 per cent. mentioned above we could not place any in the first two classes, and only 12 per cent. among those who had done fairly well, while the remaining 88 per cent. must be classed as "done badly." Of the rest the figures read, very well, 22 per cent.; well, 35 per cent.; fairly well, 25 per cent.; and badly, 18 per cent. We now come to the most startling fact of all, viz., that of those who did not enter purely by their own wish no less than 75 per cent. were sons of medical men. At first one would imagine that these latter at least would recognise the necessity of personal interest on the part of the student in their profession, but there are probably two chief causes for this strange condition. In the first place, a man who has spent his whole life in the medical profession, and whose interests are completely centred in it, would for no other reason wish his son to enter a profession which has been his sole object in life, and may be he knows little or nothing of any other. Secondly, many enter their sons merely as a matter of business, with a view of continuing their practices, and "keeping up the old name." When the sons are ready and willing to do so, nothing could be more desirable, but surely it is a great mistake to imagine that a medical practice can be perpetuated in the same manner as a public-house or a tripe-shop.

Many will doubtless contend that a brilliant academical course is not necessarily followed by a successful professional career, which we must fully admit. At the same time everyone will acknowledge that it must be extremely exceptional for an unsuccessful student to become a successful practitioner, and this article deals only with one cause of failure in the profession. We have been fortunate enough to obtain the opinions of some of the most eminent and successful medical men upon this point. They almost unanimously assert that they entered the profession entirely by choice and fully endorse the subject of this article. These include, amongst others, Sir Henry Thompson, Mr. Jonathan Hutchinson, Dr. Quain, Sir Alfred Garrod, and Sir Morell Mackenzie.

We cannot do better than conclude with an extract from a letter which we have seen from that most successful of surgeons, Mr. Lawson Tait. He says: "The conditions under which various men have entered the profession have struck me as leading up to a division of them into two very hard and fast groups. Those who have entered the profession by necessity, more or less, and those who have entered it by an earnest liking for it. *Of the former I do not know a single instance of marked success*, whilst every successful man whom I have known has been an interesting example of the latter."

